# Reaction norm analysis reveals rapid shifts toward delayed maturation in harvested Lake Erie yellow perch (*Perca flavescens*)

**DOI:** 10.1111/eva.12764

**Published:** 2019-01-30

**Authors:** Davíð Gíslason, Mikko Heino, Beren W. Robinson, Robert B. McLaughlin, Erin S. Dunlop

**Affiliations:** ^1^ Department of Integrative Biology University of Guelph Guelph Ontario Canada; ^2^ Matís Ohf Reykjavík Iceland; ^3^ Department of Biology University of Bergen Bergen Norway; ^4^ Institute of Marine Research Bergen Norway; ^5^ Evolution and Ecology Program International Institute for Applied Systems Analysis Laxenburg Austria; ^6^ Institute of Oceanography National Taiwan University Taipei Taiwan; ^7^ Aquatic Research and Monitoring Section Ontario Ministry of Natural Resources and Forestry Peterborough Ontario Canada

**Keywords:** fisheries, Laurentian Great Lakes, life history, probabilistic maturation reaction norms

## Abstract

Harvested marine fish stocks often show a rapid and substantial decline in the age and size at maturation. Such changes can arise from multiple processes including fisheries‐induced evolution, phenotypic plasticity, and responses to environmental factors other than harvest. The relative importance of these processes could differ systematically between marine and freshwater systems. We tested for temporal shifts in the mean and within‐cohort variability of age‐ and size‐based maturation probabilities of female yellow perch (*Perca flavescens* Mitchill) from four management units (MUs) in Lake Erie. Lake Erie yellow perch have been commercially harvested for more than a century, and age and size at maturation have varied since sampling began in the 1980s. Our analysis compared probabilistic maturation reaction norms (PMRNs) for cohorts when abundance was lower and harvest higher (1993–1998) to cohorts when abundance was higher and harvest lower (2005–2010). PMRNs have been used in previous studies to detect signs of evolutionary change in response to harvest. Maturation size threshold increased between the early and late cohorts, and the increases were statistically significant for the youngest age in the western MU1 and for older ages in the eastern MU3. Maturation envelope widths, a measure of the variability in maturation among individuals in a cohort, also increased between early and late cohorts in the western MUs where harvest was highest. The highest rates of change in size at maturation for a given age were as large or larger than rates reported for harvested marine fishes where declines in age and size at maturation have been observed. Contrary to the general observation of earlier maturation evolving in harvested stocks, female yellow perch in Lake Erie may be rapidly evolving delayed maturation since harvest was relaxed in the late 1990s, providing a rare example of possible evolutionary recovery.

## 1 INTRODUCTION

Changes in the life histories of commercially harvested fish populations can influence recruitment, population dynamics, and yield (Law & Grey, 1989). Maturation traits, such as age and size at maturation, have declined in many commercially harvested stocks of marine fish (Devine, Wright, Pardoe, & Heino, 2012; Heino, Díaz Pauli, & Dieckmann, 2015; Jørgensen et al., 2007; Sharpe & Hendry, 2009) and in some freshwater fish stocks (Dunlop, Shuter, & Dieckmann, 2007; Feiner et al., 2015). Earlier maturation at smaller size is an important change because, by reducing fecundity, it can decrease population productivity and yield (Dunlop, Eikeset, & Stenseth, 2015; Eikeset, Richter, Dunlop, Dieckmann, & Stenseth, 2013; Kuparinen, Stenseth, & Hutchings, 2014; Law & Grey, 1989). Sudden large declines in age at maturation may also signal an impending population collapse (Olsen et al., 2004; Trippel, 1995), underscoring the importance of monitoring maturation dynamics and understanding their drivers.

Harvest can influence age and size at maturation in wild populations through at least three mechanisms whose effects on maturation may require different management responses. First, a strong harvest that lowers density sufficiently to reduce intraspecific juvenile resource competition can enhance juvenile growth rate permitting earlier maturation at smaller size (Trippel, 1995). Plastic developmental responses to reduced juvenile density over generations should rapidly reverse as fish density increases. Second, strong size‐selective harvest that removes larger and older individuals will skew the population age‐ and size‐structure toward juveniles (Jørgensen et al., 2007). These demographic changes should also be quickly reversible by reducing the intensity or size selectivity of harvest. Third, strong persistent harvest of a population containing additive genetic variation in maturation tendency may generate an evolutionary response toward earlier age and smaller size at maturation (Grift, Rijnsdorp, Barot, Heino, & Dieckmann, 2003; Heino & Dieckmann, 2008; Heino, Dieckmann, & Godo, 2002; Law & Grey, 1989; Rijnsdorp, 1993). The reversibility of fisheries‐induced evolution (FIE) of maturation depends on the additive genetic variation remaining after harvest ceases and on other sources of selection acting on maturation (Dunlop, Enberg, Jørgensen, & Heino, 2009; Kuparinen & Hutchings, 2012). Plastic developmental, demographic and evolutionary effects on maturation in response to harvest are not mutually exclusive, and delineating their effects is challenging but important because of their management consequences (Heino & Godø, 2002).

Probabilistic maturation reaction norms (PMRNs) are used to assess the potential for evolutionary responses in maturation over time within a population by statistically accounting for common effects of variation in growth and demographic structure on age and size at maturation (Barot, Heino, O’Brien, & Dieckmann, 2004; Heino et al., 2015; Heino & Dieckmann, 2008). PMRNs express the mean probability that an immature individual that has survived and grown to a given age and size will mature at a future time (Heino, Dieckmann, & Godø, 2002). Earlier or later maturation are, respectively, revealed by downward or upward shifts in the age‐specific lengths at 50% maturation probability (Lp50) as the underlying factors that regulate the probability of maturation change over generations in a population (Heino & Dieckmann, 2008; Kuparinen & Merilä, 2007). PMRNs can shift as a result of genetic changes in size or age at maturation in a population, but might also shift as a result of phenotypic plasticity in other unmeasured factors that generate correlated maturation responses (Kraak, 2007).

Many marine fish populations under persistent harvest exhibit reduced age and size at maturation in addition to declining abundance over time (Devine et al., 2012; Heino et al., 2015; Sharpe & Hendry, 2009; Trippel, 1995). By comparison, changes in age and size at maturation in freshwater populations have been investigated less frequently (Dunlop et al., 2007; Dunlop, Shuter, & Ridgway, 2005; Feiner et al., 2015; Haugen & Vøllestad, 2001; Kokkonen, Vainikka, & Heikinheimo, 2015; Wang, Höök, Ebener, Mohr, & Schneeberger, 2008). Whether freshwater fish stocks would generally differ from marine stocks in their responses to harvest remains uncertain, given potential differences between fisheries (e.g., gear, fishers behavior, market forces), management approaches (e.g., stocking), data availability (e.g., length of time series), stock characteristics (e.g., population size, gene flow), and environmental variation (e.g., due to eutrophication and invasive species). For example, many freshwater systems have been intensively stocked with fish, have undergone substantial changes due to invasive species, and have experienced significant nutrient inputs from human development and agriculture, all of which could interact with or mask underlying evolutionary responses due to harvest (Dunlop, Feiner, & Höök, 2018). In addition, freshwater fish populations are typically much smaller than commercially important marine populations and less subject to immigration because of reduced connectivity among lake populations, which could alter additive genetic variation. Lastly, the smaller geographic scale of spatially isolated lakes increases the likelihood that stochastic environmental effects might favor greater plasticity in maturation, especially in temperate lakes with strong seasonal ecological effects. All of these factors could influence evolutionary and management responses to harvest in ways that differ from marine systems where some of the most prominent examples of fisheries‐induced evolution have arisen (Mollet, Kraak, & Rijnsdorp, 2007; Olsen et al., 2005; van Walraven, Mollet, van Damme, & Rijnsdorp, 2010).

One of the largest freshwater fisheries in the world is for yellow perch (*Perca flavescens* (Mitchill)) in Lake Erie, North America (Poste, Hecky, & Guildford, 2011), which provides a unique and valuable opportunity to examine the effects of harvest on maturation in a freshwater fish. Yellow perch have been harvested commercially and recreationally here for over a century (Brenden, Brown, Ebener, Reid, & Newcomb, 2013), and over this time, the intensity of harvest and abundance have varied considerably. For example, the proportion of the population harvested annually (annual exploitation rate or harvest proportion; µ) has been as high as 0.6 since 1975 (Baldwin, Saalfeld, Dochoda, Buettner, & Eshenroder, 2009; Belore et al., 2016). Yellow perch have a life history characterized by medium body size, high fecundity, high juvenile mortality, and considerable variability in cohort strength, similar to many marine fishes (Winemiller & Rose, 1992). Earlier analyses of PMRNs within and among yellow perch populations of the Great Lakes from 1975 to 2010 suggested that persistent harvest of Lake Erie yellow perch might have contributed to earlier maturation, while reductions in harvest of yellow perch in Lakes Huron and Michigan might have allowed recovery of delayed maturation (Feiner et al., 2015). However, this previous research aggregated maturation data over a 10‐year period due to sample size limitations, whereas recent work indicated that annual mean maturation varied dramatically at scales below a decade and this could not be attributed to plastic developmental responses to harvest‐induced density‐ and growth‐dependent effects on maturation schedules (Gíslason, McLaughlin, Robinson, Cook, & Dunlop, 2017). For example, mean length and age at 50% maturity varied from 15.0 to almost 18.5 cm, and from 1.5 to 3 years of age, over an 9‐year period (1996–2004). Feiner et al. (2015) were also unable to consider the probability of maturation at age 2, the earliest age at which yellow perch mature, due to lack of data. In this study, we were able to expand the earlier analysis by Feiner et al. by including additional agency data. This allowed us to reduce the degree of data aggregation from 10 to 6 years, include age 2 fish, and separate the data spatially to compare maturation trends among management units within Lake Erie where harvest intensity has varied and yellow perch display evidence of genetic differentiation (Sepulveda‐Villet, Stepien, & Vinebrooke, 2011; Sullivan & Stepien, 2015).

**Figure 1:**
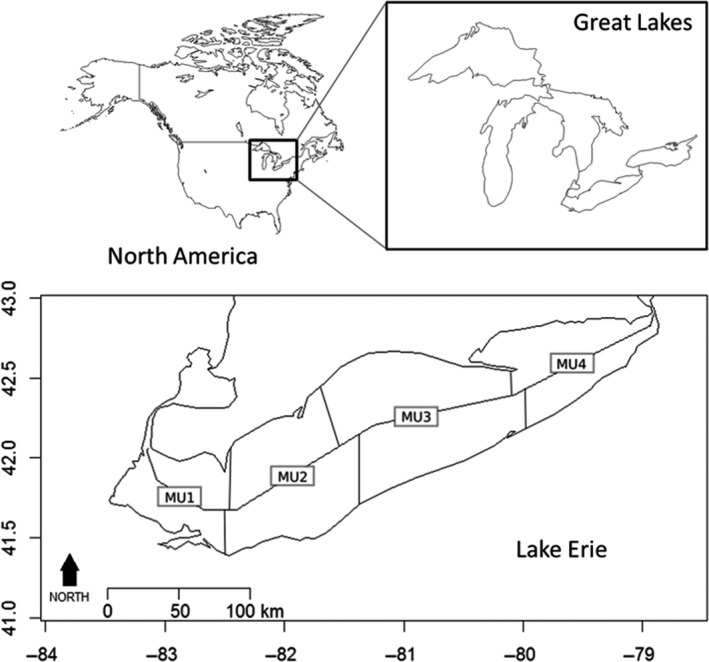
Map of Lake Erie showing the four management units (MUs) for yellow perch numbered from west to east. The black square in the upper left insert shows the location of the Laurentian Great Lakes in North America (composed in R 3.0.2 using maps)

**Figure 2:**
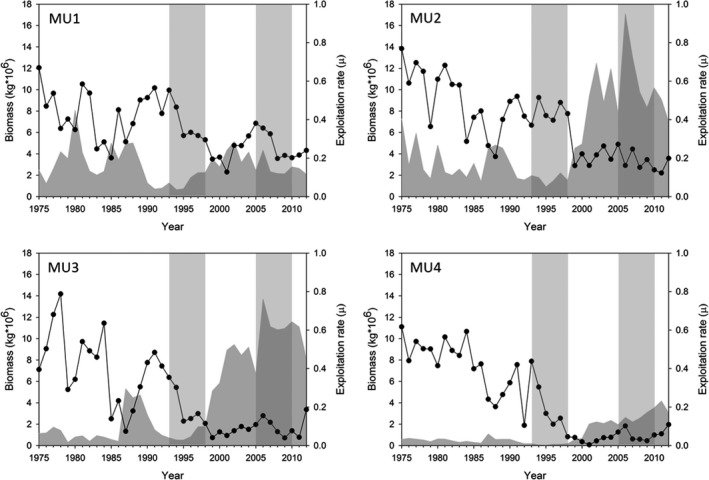
Population biomass and the proportion of biomass harvested (exploitation rate) of yellow perch in Lake Erie management units (MUs) from 1975 to 2012 (Belore et al., 2014). The gray filled area shows the biomass of age 2 and older fish and the exploitation rate is shown as a black line. Light gray columns show the contrasted early (1993–1998) and late (2005–2010) cohort sets each consisting of six sequential cohorts that were combined within the set to estimate a probabilistic maturation reaction norm

Lake Erie’s shallow depth and high nutrient input drive exceptional productivity that supports the large commercial fishery. The lake consists of three basins that create a gradient in nutrient loading, productivity, and water depth. The mesotrophic western basin is the shallowest (mean depth 7.5 m, max. depth 19 m), the oligomesotrophic central basin has intermediate depth (mean 18.3 m, max. 25 m), and the oligomesotrophic eastern basin is deepest (mean 24 m, max. 64 m; Allinger & Reavie, 2013). The yellow perch fishery in Lake Erie is managed under a bi‐national agreement between Canada and the United States that recognizes four geographic management units (MU1–MU4 from west to east; the middle basin includes MU2 and MU3, Figure 1) based on their unique biota, hydrological properties, and evidence of persistent population genetic differentiation in yellow perch and other fishes across the MUs (Sepulveda‐Villet et al., 2011; Sullivan & Stepien, 2015). Population abundance and catch decline from west to east (Figure 2). Since 1975, the total commercial catch of yellow perch has been highest in MU2, followed by MU1, MU3, and lowest in MU4 (Belore et al., 2014). The Ontario commercial yellow perch fishery has been managed at the MU scale by an Individual Transferrable Quota system (ITQ) since 1984 (Brenden et al., 2013).

We used the spatial structure of the Lake Erie management units and 21 years of data on life history and harvest to investigate whether PMRNs for female yellow perch have changed over time in response to spatial and temporal variation in harvest intensity. We estimated PMRNs at a historic and more recent time period in each MU and compared these to examine three possible changes in age and length at maturation that could reveal insights into the influence of harvest on maturation schedule. First, we tested if the PMRN midpoints (the estimated relationship between Lp50 and age) for a set of six cohorts born from 2005 to 2010 (late cohort set), a period when harvest was low and abundance was high, differed from the PMRN midpoints from a set of six cohorts born from 1993 to 1998 (early cohort set), a period when harvest was high and abundance was low. Second, we tested whether the envelope width of the PMRN changed between the early and late cohort sets. The width of the PMRN is expressed as the distance between mean lengths at 25% and 75% probability of maturation for a given cohort. That width characterizes genetic variation in maturation reaction norm midpoints among individuals (Heino & Dieckmann, 2008) and environmental variance generated by factors other than growth (Olsen et al., 2004). Strong selection on size could reduce additive genetic variance in maturation and therefore reduce the PMRN envelope width. Consistent temporal changes in PMRN midpoints and envelope width would suggest that maturation had evolved and that variation influencing the opportunity for and response to selection had changed in the population, providing insights into whether harvest has been an important source of selection on maturation in yellow perch. Third, we compared standardized measures of phenotypic change in Lake Erie yellow perch with measures for harvested marine fishes to evaluate whether maturation responses to harvest in this large freshwater fishery were similar in magnitude to responses reported for harvested marine fishes.

## 2 METHODS

For our analyses, we treated the four yellow perch MUs as statistically independent units, even though the spatial population structure of yellow perch in Lake Erie remains unresolved. We took this approach for five reasons. First, current information about the spatial structure of physical and chemical conditions, and about the population differentiation of yellow perch in Lake Erie, supports the use of separate management units. Second, stock assessments and harvest have been uniquely determined for and regulated at the MU scale since 1984 (Figure 2). Third, estimating PMRNs for each MU provides an opportunity to directly compare changes in PMRN characteristics and variation in harvest among MUs. Fourth, aggregating maturation data across the four MUs could limit our ability to distinguish the effects of changing harvest over time from the confounding effects of other temporal factors that might influence yellow perch maturation in Lake Erie. For an analysis of data aggregated across MUs, there is no unharvested population available to serve as a reference over the same interval. Fifth, conclusions obtained when aggregating data across the entire lake could be unreliable, due to bias that could arise because yellow perch abundance (Figure 2) and numbers of yellow perch sampled in index surveys are greater for the western MUs than for the eastern MUs. Analyzing the MUs separately could have one potentially important drawback. If no temporal change in PMRN features is observed among the MUs, we will not be able to distinguish whether harvest effects are absent or whether the population structure is more homogeneous than expected.

Our analyses were completed using spatially and temporally referenced data on age, length, sex, and maturation status (mature/immature) of individual Lake Erie yellow perch from cohorts born from 1991 to 2010. In Lake Erie, female yellow perch have a mean generation time of 4.0 years but can mature from ages 2 to 4 years and live to 14 years. We only analyzed females because changes in their maturation schedule are expected to more strongly affect population dynamics due to size‐dependent fecundity than that of males. Data were obtained from the Lake Erie partnership index fisheries survey database maintained by the Lake Erie Management Unit of the Ontario Ministry of Natural Resources and Forestry (OMNRF). The database was created through a partnership between OMNRF and the Ontario Commercial Fisheries Association to assist with stock assessment and management. Survey data were collected using fisheries‐independent fall gill net surveys in the Canadian waters of Lake Erie (employing monofilament nets composed of 25 panels of 14 stretched mesh sizes: 32, 38, 44, 51, 57, 64, 70, 76, 89, 102, 114, 127, 140, and 152 mm: OMNRF & OCFA, 2016). From 1991 to 2010, the number of gangs fished annually varied from 58 to 144 (mean = 125) and covered all four MUs. The full‐size ranges of age 2 and older yellow perch were sampled, but only the larger size ranges of age 1 individuals were likely sampled (A. Cook, OMNRF, Wheatley, ON, Canada, personal communication).

Our tests of possible changes in age and length at maturation entailed comparisons of cohort sets from 1993 to 1998 and from 2005 to 2010. These two sets were selected for comparison because they provided the greatest feasible time contrast and the greatest statistical power to detect possible evolutionary changes within the time series data available. In addition, these two sets contrasted an early period when abundance was lower and harvest higher with a later period when abundance was higher and harvest lower. Combining six cohorts within each set was the minimum required to ensure adequate sample sizes to estimate PMRNs (Table 1). The early cohort set began at 1993, rather than 1991, because the method used to estimate PMRNs (outlined below) requires maturation data on cohorts preceding the focal cohorts.

**Table 1:** Sample sizes of immature (I) and mature (M) female yellow perch aged 2–5 years from Lake Erie that were used to estimate PMRNs for early (1993–1998) and late (2005–2010) cohort sets for each MU

Cohort year	Age 2		Age 3		Age 4		Age 5	
	I	M	I	M	I	M	I	M
Individual management units—time periods								
MU 1, 1993–1998	824	51	278	1,046	77	694	12	340
MU 1, 2005–2010	295	4	47	23	6	29	0	9
MU 2, 1993–1998	817	174	182	2,047	12	1,199	1	545
MU 2, 2005–2010	176	6	60	99	2	242	0	135
MU 3, 1993–1998	269	28	234	1,417	46	1,740	12	1,075
MU 3, 2005–2010	203	11	207	127	36	197	6	234
MU 4, 1993–1998	102	12	88	329	15	526	7	414
MU 4, 2005–2010	446	22	177	429	64	286	11	237

Probabilistic maturation reaction norms represent probabilities of maturing in a future specified interval as a function of age and size for specific cohorts or combined sets of cohorts of individuals. We estimated PMRNs using the demographic estimation method of Barot et al. (2004) because it was not possible to identify individuals spawning for the first time with the available data (Heino & Dieckmann, 2008). PMRNs were estimated for each MU separately and for each time period or cohort as described below (sample sizes in Table 1). We characterized the PMRN using age‐specific estimates of mean length with 50% probability to mature in the next year (Lp50) and the 25% to 75% maturation envelope width based on age‐specific estimates of Lp25 and Lp75. A value of one was added to age because female yellow perch were sampled in the fall prior to spring spawning (Feiner et al., 2015); size was not adjusted because no growth is expected between the fall and the spring spawning (Farmer, Marschall, Dabrowski, & Ludsin, 2015).

Probabilistic maturation reaction norms were estimated as:

(1) $$m\left(a,s\right)=\frac{o(a,s)-o(a-1,s-\Delta s(a))}{1-o(a-1,s-\Delta s(a))}$$

where *m*(*a*,*s*) is the probability of an individual maturing at age *a* and size *s*, ∆*s* is the change in size (total length) from age *a*−1 to *a,* and *o*(*a,s*) is the proportion of mature individuals at a given age and size (the maturity ogive).

Calculation of the PMRNs (Equation 1) required statistical estimates of the growth increments (∆*s_a_*) and age‐specific maturity ogives (*o*(*a,s*)) from two immediately preceding cohorts. Age‐ and cohort‐specific growth increments were estimated by predicting average length at age using a linear growth model relating length as the dependent variable with both age and cohort as factors:

(2) length∼age+cohort

Maturity ogives, a curve representing the proportion of mature fish by age or size, were estimated using logistic regression. A set of logistic models was first created that related the proportions of mature (*o*) and immature (1−*o*) individuals as the dependent variable with combinations of age, size, cohort, and their first order interactions as the maximal model. The best model was then chosen based on Akaike’s information criterion (AIC; Table 2).

**Table 2:** Akaike’s information criterion (AIC) comparison of logistic ogive models relating the probability of being mature to age (A), total length (L) and cohort year (C) for 2–5‐year‐old female yellow perch from cohorts born between 1991 and 2010

Model	AIC	∆AIC	*R* ^2^
A + L + C + A × L + A × C + L × C	14,477	0	0.71
A + L + C + A × C + L × C	14,536	59	0.71
A + L + C + A × C	14,673	196	0.71
A + L + C + L × C	14,744	267	0.71
A + L + C + A × L	15,052	575	0.70
A + L + C	15,175	698	0.70
L + C	15,255	778	0.69
A + L	16,327	1,850	0.66
Length	16,423	1,946	0.66
A + C	20,434	5,957	0.55
Age	22,479	8,002	0.49
Cohort	31,250	16,773	0.17

Note

The best model was used for analyses of Lp50 between early (1993–1998) and late (2005–2010) cohort sets for individual management units. *R* ^2^ was estimated for logistic regression using the Nagelkerke method (Nagelkerke, 1991).

After selecting the equations for ∆*s_a_* and *o*(*a,s*), PMRNs were estimated separately for each cohort and age from 2 to 5 (Equation 1). Bootstrapping was used to generate approximate 95% confidence intervals for cohort‐ and age‐specific PMRN midpoint values (1,000 bootstrap samples from each estimate). The PMRN midpoints for the six early and the six late cohorts in each MU were estimated by combining the specified cohorts and bootstrapping with replacement within each combined cohort set. In all cases, bootstrap sampling chose individuals at random with replacement from the specified cohort and age so that the final bootstrapped sample size was the same as the original sample. The maturity ogive, growth, and PMRN were all estimated from the resampled data. Confidence intervals were derived as 2.5% and 97.5% quantiles of the resulting distributions of PMRN midpoints.

We used randomization tests to determine whether the observed relationship between mean Lp50 and age differed between the early and late cohort sets (test 1). For a given age, individuals were randomly shuffled between the early and late cohort sets while retaining the original numbers of individuals (sample sizes) observed in each cohort set. Following the randomization, Lp50 was then calculated for each cohort set, and the difference in Lp50 values between cohort sets was determined. This process was completed 999 times to obtain a distribution comprised of 999 differences calculated via randomization plus the observed difference (*N* = 1,000). The probability of obtaining an absolute difference as large or larger than the absolute value of the observed difference was estimated using this distribution. The process was repeated for each of the age classes. In test 2, we tested for changes in PMRN envelope width between early and late cohort sets using the same randomization testing procedure just described, except in this case we focused on the difference in the envelope width (Lp75−Lp25) between cohort sets, rather than the difference in Lp50.

Changes in PMRN midpoints and PMRN envelope width over time were quantified in three ways to facilitate comparisons with other studies: percent change, and standardized change expressed in darwin and haldane units (Gingerich, 2001). Percent change was calculated as Lp_2_/Lp_1_−1, where Lp*_t_* represents PMRN midpoints or envelope width in late (2005–2010) and early cohorts (1993–1998). The standardized change in darwins (*d*) was calculated as ln(*x*_2_/*x*_1_)/∆*t*10^−6^, where *x*_1_ is the Lp50 value for the early period, *x*_2_ is the Lp50 value for the later cohorts, and ∆t is the number of years between the two cohort sets. The standardized change in haldanes (*h*) was calculated as (ln *x*_2_/Sp ln *x* − ln *x*_1_/Sp ln *x*)/∆*t*_g_, where *x*_1_ and *x*_2_ are the sample means for age‐specific Lp50 for the early and late cohort sets, respectively, Sp ln *x* was the pooled standard deviation of ln *x*_1_ and ln *x*_2_, and ∆*t*_g_ is the time difference expressed in generations between the two cohort sets. Generation time *t*_g_ was estimated as:

(3) $$t_{g}\approx \frac{\sum_{t}^{t_{\max}}t\times S_{t}\times M_{t}\times W_{t}}{\sum_{t}^{t_{\max}}S_{t}\times M_{t}\times W_{t}}$$

where *t*_max_ is maximum age, *S_t_* is numbers at age *t*,* M_t_* is maturation ogive, and *W_t_* is the average mass at age *t* (Devine et al., 2012).

All calculations and tests were performed independently for each MU using R 3.3.1 (R Core Team, 2014).

## 3 RESULTS

There was spatial variation in stock and harvest dynamics among yellow perch from the four Lake Erie MUs. Biomass increased significantly from the early (1993–1998) to the late (2005–2010) periods in MU2 and MU3, to a lesser extent in MU4, and minimally in MU1 (Figure 2). Over this interval, exploitation rate was relatively high but variable in MU1, but declined in MU2–4. On average, the decline in exploitation rate between cohort periods was more abrupt for MU2 than for MU3 and MU4 where the declines were similar.

**Figure 3:**
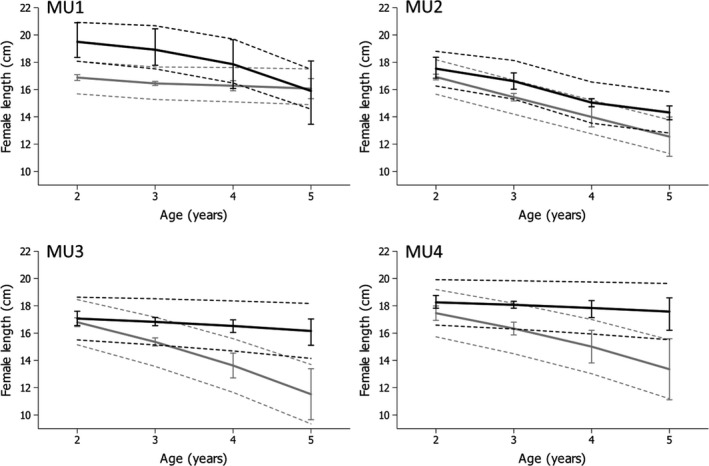
Probabilistic maturation reaction norms (PMRNs) estimated for female yellow perch for each of four Lake Erie management units during the early period (1993–1998: gray line) and the late period (2005–2010: black line). Solid lines show the sizes at which the probability to mature is 50% (the PMRN midpoint). 95% confidence intervals for PMRN midpoints are shown as error bars at each age. Thin dotted lines depict the maturation envelope where the probability of maturing increases from 25% (lower lines) to 75% (upper lines)

Probabilistic maturation reaction norm midpoints were higher in the late cohort set than in the early cohort set for at least some age classes across all MUs except MU4 (Table 3, Figure 3). However, the age classes where elevated midpoint values were expressed in the 2005–2010 cohort set differed between MUs. The PMRNs for younger ages were visibly shifted upwards (generating a negative slope in the later cohort set) in MU1, whereas the PMRNs for older ages were visibly shifted upwards in MU2 and MU3 (Figure 3). Statistically significant changes in age‐specific Lp50 maturation probabilities were always positive (Table 3), ranging from 9.6% to 40% (length increases of 1.5–4.6 cm). No statistically significant changes in age‐specific maturation probabilities were detected for MU4 (Table 3).

**Table 3:** Age‐specific estimates of the length at which the probability of maturation in female Lake Erie yellow perch was 50% for each management unit (MU) and for cohort sets from early (1993–1998) and later (2005–2010) periods

Area	Age (years)	Length (cm) at 50% maturation probabilities						
		1993–1998 (cm)	2005–2010 (cm)	Change				*p*
				(cm)	(%)	(kd)	(*h*)	
MU1	2	16.9	19.5	2.6	15.5	12.0	1.56	**0.002**
	3	16.5	18.9	2.5	15.0	11.6	1.34	0.07
	4	16.3	17.9	1.6	9.7	7.7	0.6	0.5
	5	16.1	15.9	−0.2	−1.1	−0.9	−0.1	0.8
		Mean			9.8	7.6	0.9	
MU2	2	16.9	17.5	0.6	3.6	3.0	0.6	0.3
	3	15.4	16.6	1.2	7.6	6.1	1.23	0.2
	4	14.0	15.0	1.1	7.6	6.1	0.91	0.1
	5	12.6	14.3	1.8	14.1	11.0	0.89	**0.02**
		Mean			8.2	6.5	0.9	
MU3	2	16.8	17.1	0.3	1.6	1.3	0.3	0.3
	3	15.4	16.8	1.5	9.6	7.6	2.43	**0.05**
	4	13.6	16.5	2.9	21.3	16.1	2.01	**<0.001**
	5	11.5	16.2	4.6	40.3	28.2	1.5	**<0.001**
		Mean			18.2	13.3	1.7	
MU4	2	17.5	18.3	0.8	4.5	3.7	0.8	0.9
	3	16.3	18.1	1.7	10.6	8.4	1.01	0.9
	4	15.0	17.8	2.8	18.9	14.4	1.2	0.4
	5	13.4	17.6	4.2	31.6	22.9	1.2	0.9
		Mean			16.4	12.4	1.01	

Note

Values are provided for each age from 2 to 5 years and for each time period. Change in Lp50 values between late and early cohorts is provided as a difference (cm), percent change, and standardized rates of change (kilo‐darwins [kd] and haldanes [*h*]). A two‐sided *p* shows the probability that the observed difference in Lp50 between time periods being observed could occur by chance based on randomization tests (*p* < 0.05 in bold). Analyses were conducted separately for each MU.

Changes in the width of the maturation envelope (Lp75−Lp25) from early to late cohort sets also varied among MUs. Envelope width increased with time in MU1 and MU2 and decreased in MU3 and MU4 (Table 4, Figure 3). Age‐specific maturation envelope width increased significantly only for fish aged 2 (25%) and 3 years (33%) in MU1, narrowed for ages 2–5 (by 5.4%–7.4%) in MU3, and did not change significantly over time for any other ages in MU1 and MU3, or for any ages in the other MUs (Table 4).

**Table 4:** Mean width of the maturation envelope (cm) between the age‐specific estimates of the length at which probability of maturation was 0.25 (Lp25) and the length at which probability of maturation was 0.75 (Lp75) for female yellow perch in Lake Erie

Area	Age (year)	Maturation envelope width: Lp75−Lp25				
		1993–1998 (cm)	2005–2010 (cm)	Change		*p*
				(cm)	%	
MU1	2	2.3	2.9	0.5	24.5	**<0.001**
	3	2.4	3.1	0.8	32.8	**<0.001**
	4	2.5	3.2	0.7	28.3	0.1
	5	2.6	2.9	0.3	11.5	0.2
MU2	2	2.5	2.5	0.02	0.8	0.7
	3	2.5	2.8	0.4	14.1	0.6
	4	2.5	3.0	0.6	22.3	0.6
	5	2.5	3.0	0.6	22.8	0.6
MU3	2	3.3	3.1	−0.2	−5.4	**0.05**
	3	3.6	3.4	−0.2	−6.4	**0.03**
	4	3.9	3.7	−0.3	−6.9	**0.02**
	5	4.4	4.0	−0.3	−7.4	**0.01**
MU4	2	3.5	3.3	−0.1	−3.8	0.2
	3	3.7	3.5	−0.2	−4.3	0.2
	4	4.0	3.8	−0.2	−4.3	0.2
	5	4.3	4.1	−0.2	−4.6	0.1

Note

Values were calculated for each age from 2 to 5 years for cohort sets from early (1993–1998) and later (2005–2010) periods in each MU. The change in envelope width between late and early cohorts is provided in cm and as percent change. A two‐sided *p* shows the probability that the observed difference in envelope width between time periods being observed could occur by chance based on randomization tests (*p* < 0.05 in bold). Analyses were conducted separately for each management unit (MU).

Phenotypic rates of change in Lp50 from the early (1993–1998) to the late (2005–2010) cohort sets were high. Generation time was estimated to be 4.0 years (95% CI: 3.7–4.3 years). Over all ages and MUs, standardized rates of change ranged from −0.9 to 28.2 kilodarwins and −0.1 to 2.4 haldanes, depending on age and MU (Table 3). The highest rates of change in length at maturation were all positive and observed for fish of ages 2 and 3 years in MU1 and ages 3 and 5 years in MU3.

## 4 DISCUSSION

Our findings suggest that maturation schedules in harvested Lake Erie yellow perch have evolved toward delayed maturation over a period from 1993 to 2010, corresponding to approximately five generations. This outcome stands in contrast to the dominant pattern found for commercially harvested marine fish stocks, which typically display trends toward earlier maturation and smaller size at maturation (Devine et al., 2012; Heino et al., 2015; Sharpe & Hendry, 2009; Trippel, 1995). Size at maturation tended to increase in all MUs, with statistically significant increases detected for all MUs but MU4. In addition, variation in the probability of maturation within cohort sets increased from 1993 to 2010 in MU1 and declined in MU3. All of these changes occurred during a period when harvest pressure was declining and population biomass was increasing, suggesting Lake Erie yellow perch could represent a rare example of possible evolutionary recovery.

Our findings reveal how inferences from shifting PMRNs can be shaped by the methods used to study maturation in harvested fish populations. Feiner et al. (2015) report a general decrease in female yellow perch PMRN midpoints in the central basin and little change in female midpoints in the western basin of Lake Erie, in contrast to the general increases observed in our study. This discrepancy could be a consequence of at least three features that distinguish our study from that of Feiner et al. (2015). First, the Feiner et al. analysis compared PMRNs midpoints over a longer, 35‐year period (1975–2010) than our study (1993–2010). During the early portion of Feiner’s time series (and as shown in our Figure 2), exploitation rates were much higher (by about 2–3 times) than they were during the years considered in our study. Second, our study used a finer temporal resolution when estimating PMRNs (aggregating cohorts over a 6‐year period), whereas Feiner et al. aggregated data over a 10‐year period (comparing cohorts among three decades: 1980–1989, 1990–1999, and 2000–2009). Third, we were able to estimate PMRN midpoints for age 2 fish from the data obtained from index gill net surveys conducted by the OMNRF. The gill net surveys were likely more effective at sampling smaller, younger fish than the trawling survey data available to Feiner et al. The differences in ages recruited to the fishing gear used between studies could be particularly salient because yellow perch in Lake Erie start to mature at age 2, with the majority of individuals becoming mature by age 3 (Gíslason et al., 2017). Some statistically significant changes in maturation were observed for age 2 in MU1 (PMNR midpoint) and MU3 (PMNR width). The important message is that data features can subtly influence estimation of PMRNs and ultimately interpretations of how maturation might be shaped by harvest.

Despite these methodological differences, our study also shares an important consistent interpretation with Feiner et al. (2015). The size and age at maturation can increase in yellow perch as harvest is reduced. In their comparison of yellow perch populations across different Great Lakes, Feiner et al. (2015) concluded that yellow perch PMRNs changed little over decades in Lake Erie where commercial harvest had continued, but increased over decades in lakes Huron and Michigan where harvest had been substantially reduced (including a complete closure in Lake Michigan). Our analyses for Lake Erie yellow perch similarly show maturation tended to be delayed from early to late cohort groups that corresponded with a general reduction in harvest in all MUs (Figure 2). These results support the idea that under some conditions, delayed maturation in fish stocks can follow reductions in harvest, over a relatively short time interval.

A shift toward delayed maturation in a wild harvested population is an exciting result, suggesting that changes in size‐dependent mortality could exert selection on maturation in either direction depending on the circumstances (Devine & Heino, 2011). Reversing an evolutionary decline in age and size at maturation may be slow when harvest is substantially reduced or ceases, for a number of reasons (Enberg, Jørgensen, Dunlop, Heino, & Dieckmann, 2009; Kuparinen & Hutchings, 2012). Natural selection arising from the positive female body size–fecundity relationship that could favor increased age and size at maturation may be weak or nonexistent when populations are at very low abundance (Swain, Sinclair, & Hanson, 2007). Selection from intense harvest may be much stronger than natural selection favoring delayed maturation because of the fecundity advantage to large females and direct mortality cost on lifetime reproductive success, leading to a quicker pace of evolution during harvesting, but a slower rate of reversal after harvest ceases (Dunlop, Heino, & Dieckmann, 2009). Alternative life history strategies with equivalent lifetime reproductive outputs for different ages at maturation may also delay evolutionary responses (Kuparinen & Hutchings, 2012; Law & Grey, 1989). Further, genetic variation for maturation reduced by a sustained strong selection may limit the opportunity for selection or enhance processes involving drift (Allendorf & Hard, 2009). In principle, genetic shifts that affect many developmental, reproductive and foraging‐related traits could potentially influence growth and survival, contributing to the idea that over‐fished populations can incur a “Darwinian debt” that slows recovery after harvest ceases (Dieckmann, Heino, & Rijnsdorp, 2009). Interestingly, the only modeling study examining fisheries‐induced evolution and potential recovery in fishes native to the Great Lakes predicted faster rates of population recovery in yellow perch than in lake whitefish (*Coregonus clupeaformis* (Mitchill)), and Atlantic cod (*Gadus morhua* Linnaeus), a marine fish (Dunlop et al., 2015). This aligns with our observation of a more rapid recovery in Lake Erie yellow perch than might be expected based on observations from other fishes. Unfortunately, neither we nor Feiner et al. (2015) know the rate at which maturation changed when harvest first began or was considerably higher than at present, and so we cannot determine whether the rate of recovery in size at maturation as harvest is relaxed is relatively quicker than past phenotypic changes under higher harvest. In any case, our study suggests that maturation traits in yellow perch are capable of recovering in relatively few generations and at a time scale relevant to fishery management.

The delayed maturation we observed for female yellow perch could also result from specific attributes of the gear used in the fishery or other factors that influence observed maturation patterns. Many marine fishes are harvested by trawling, which tends to impose sigmoidal size selectivity where the probability of capture increases with increasing size and immature fish are included in the harvest (Dunlop, Heino et al., 2009; Enberg et al., 2012; Jørgensen, Ernande, & Fiksen, 2009; Kuparinen, Kuikka, & Merilä, 2009; Mollet, Poos, Dieckmann, & Rijnsdorp, 2016). This form of size selectivity can generate strong selection against larger and later maturing individuals (Hutchings, 2009; Jørgensen et al., 2009) and contribute to the common trend in marine fish of reduced age at maturation over time (Jørgensen, 1990; Kuparinen et al., 2009). In contrast, freshwater fishes like yellow perch are commonly caught using gill netting and angling, which can exert dome‐shaped size‐selective harvest when intermediate sizes have the highest probability of capture. At low harvest intensity, dome‐shaped size selection can favor delayed maturation when fish are able to grow through the harvested size range (Hutchings, 2009). Conversely, when harvest intensity is high, dome‐shaped size selectivity can favor earlier maturation when only few fish can grow through the targeted size range (Hutchings, 2009; Jørgensen et al., 2009). In addition, other traits related to maturation, such as growth rate, can evolve in response to changes in harvest and have a correlated influence on maturation (Enberg et al., 2012). These examples showcase the diversity of ways in which the intensity and methods of harvest can influence the evolution of maturation.

The selectivity and intensity of yellow perch harvest are additionally complicated in Lake Erie because commercial and recreational fishers both contribute to harvest and their contributions to total harvest vary annually. Commercial fishers use gill nets, whereas the recreational fishers rely on angling. Commercial fishers likely dominate the selectivity and intensity of total harvest. Recreational anglers accounted for only 17%–30% of the total annual catch between 1995 and 2010 (Belore et al., 2016). However, the influence of recent commercial harvest on maturation may be easing because commercial harvest intensity has steadily declined since the mid‐1970s (Figure 2). In addition, the selectivity of commercial gill nets may be weak because from 1996 to 2008 the mean length of females taken in the commercial catch has been greater than the mean length at which 90% of females become mature (Gíslason, University of Guelph, personal observation). The selectivity of recreational angling is more speculative. Angling can exert sigmoidal size selection when many medium and large fish are taken, but this may not occur in Lake Erie because recreational anglers catch a wide range of sizes and ages of yellow perch (Belore et al., 2016). Recreational angling of yellow perch in Lake Erie has no size limits, with the exception of Pennsylvanian waters where a minimum size limit of seven inches (about 18 cm) is imposed between December and May (Brenden et al., 2013). However, all jurisdictions limit the daily allowable angling catch. So it remains unclear how recreational harvest might influence maturation, especially in light of the generally greater intensity exerted by the commercial fishery. Detailed data on the selectivity of the commercial and recreational harvests are required to evaluate how harvest selectivity as a whole may affect maturation in yellow perch.

**Figure 4:**
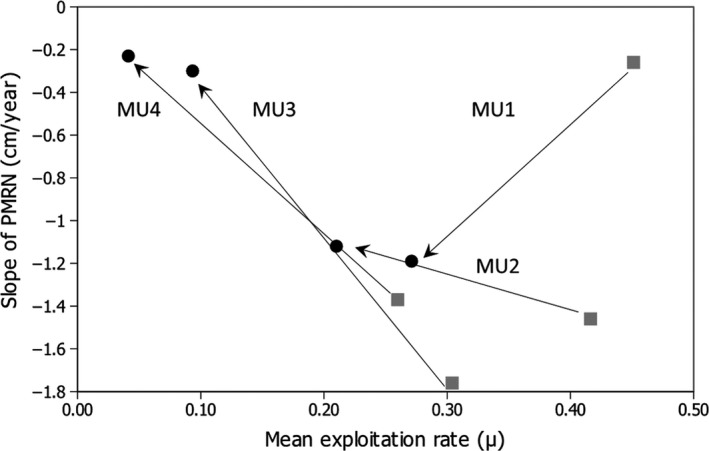
Change in slope of probabilistic maturation reaction norms (PMRNs) and of harvest from the early (gray squares) to late (black circles) cohort set for each management unit (MU). PMRN slopes were estimated by fitting a linear regression through the midpoints (i.e., length at 50% maturation probability) values (Table 3). Mean annual harvest was estimated over the interval 1985–1993 for the early cohort set and 2000–2008 for the late cohort set. The harvest intervals are offset 2 years earlier than the end, and 5 years earlier than the beginning of the actual cohort years in order to account for the effects of harvest prior to the birth years of the different age classes

We tested the effects of harvest on maturation using the four MUs of the Lake Erie yellow perch fishery and found a general pattern of larger increases (i.e., recovery) in length at maturation over time with declining harvest pressure, although age‐related responses varied among MUs. The generally lower levels of annual harvest in the eastern MU3 and MU4 suggest that these locations might provide useful contrasts to the western, more heavily harvested MU1 and MU2 (Figure 4). Perhaps because of the somewhat lower harvest pressure, yellow perch in MU3 and MU4 showed the largest shift over time from negatively sloped to near horizontal PMRNs as harvest was relaxed (although the change was not significant in MU4). This response was driven mostly by large increases in length at maturation in older ages (Figure 3) in the eastern MUs in contrast to western MUs (contrast of change in age‐5 Lp50 for MU1 & MU2 vs. MU3 & MU4, *z* = 54.4, *p* < 0.00001). These findings are consistent with the hypothesis that the lower absolute harvest in the eastern MUs enabled a faster rate of recovery of PMRNs than in western MUs. We considered but rejected the idea that the changes in slopes reflect statistical artifacts based on differences in data available on maturation in each MU. Each cohort set combined six annual cohorts of data to generate large sample sizes for each age from 2 to 5 during PMRN estimation, although sample sizes were smallest for MU4 (and may have contributed to the lack of statistical evidence of change over time there). Sample sizes were also smaller but still sufficient to estimate PMRNs for the 2000–2005 cohort set compared to the 1993–1998 cohort set (Table 1).

The temporal changes in PMRNs are challenging to interpret among individual MUs (Figure 4) because the extent to which the spatially defined MUs represent the underlying stock structure of yellow perch is unclear. There are also potential differences in specific fishing patterns (e.g., spatial location, fisher behavior, gear type), relative importance of commercial and recreational harvest, and other sources of mortality (e.g., predation) between MUs. Most harvested marine fishes analyzed to date express PMRNs with neutral or negative slopes (Heino & Dieckmann, 2008), suggesting that strong persistent size‐selective harvest may generally favor these forms of PMRNs. In theory, strong size‐selective harvest can both reduce the PMRN intercept (e.g., favor maturation at smaller size over all ages) and rotate the slope of the PMRN clockwise (e.g., favor maturation at smaller sizes in older individuals; Marty, Dieckmann, & Ernande, 2015). A negatively sloped PMRN indicates that individuals who grow slowly have a greater tendency to mature at a smaller size than fast‐growing individuals (Heino & Dieckmann, 2008). Other modeling studies have predicted little evolution of PMRN slopes in response to harvest (Dunlop, Heino et al., 2009; Eikeset et al., 2016). Superficially, the shift to a negative sloped PMRN for yellow perch in MU1 where harvest declined somewhat but remained high relative to other MUs is consistent with the effects of size‐selective harvest (Figure 4). However, it is important to note that this arose through an increase in length at maturation in younger age 2 fish, not through a reduction in length at maturation in older age classes (Figure 3), which is inconsistent with the effects of size‐selective harvest. Interpreting temporal shifts in PMRN in MU2, MU3, and MU4 seems more consistent with theory. The PMRNs rotated counter‐clockwise as harvest was relaxed (Figure 4). The challenges with providing satisfactory explanations for the different shifts in PMRNs among these few regions suggest that a variety of environmental factors and population constraints in addition to harvest could influence changes in PMRN slopes.

The changes observed in PMRN envelope width over this time period are also exciting, because minimal empirical or theoretical effort has addressed how envelope widths should evolve under harvest. Variation around the population PMRN reflects genetic variation in maturation reaction norm midpoints (Heino & Dieckmann, 2008) and environmental variance generated by factors other than growth (Olsen et al., 2004). Unexpectedly in Lake Erie yellow perch, envelope width increased over time under high harvest in MU1, particularly in the youngest age classes, and decreased under low harvest in MU3 for all age classes. These changes are inconsistent with strong size‐selective harvest depleting natural variation in a harvested population. Diverse changes in envelope width mimic a pattern observed among harvested populations where natural diversity has been lost in some (Hauser, Adcock, Smith, Bernal Ramirez, & Carvalho, 2002; Hoarau et al., 2005; Hutchinson, van Oosterhout, Rogers, & Carvalho, 2003) but not in other harvested stocks (Ruzzante, Taggart, Doyle, & Cook, 2001; Therkildsen, Nielsen, Swain, & Pedersen, 2010). Spatial differences in population genetic variation have not changed significantly in Lake Erie’s yellow perch from 2001 to 2009 (Sullivan & Stepien, 2015), suggesting that the shifts in envelope width we detected might reflect environmental effects on maturation unrelated to growth (Olsen et al., 2004). Alternatively, the reciprocal shifts in envelope width between MUs could represent uncertainty about stock structure and the movement of individuals among MUs. The theory explaining shifts in PMRN envelope width in response to harvest, environmental variation, and population structure requires further development.

It is important to acknowledge a general uncertainty about evolutionary inferences derived from changes in PMRNs involving natural populations. While the logic of PMRN analysis is compelling, maturation can be affected by factors other than size and age, such as growth history, body condition, thermal and even social aspects of the environment (Diaz Pauli & Heino, 2013; Grift, Heino, Rijnsdorp, Kraak, & Dieckmann, 2007; Morita & Fukuwaka, 2006; Morita, Tsuboi, & Nagasawa, 2009; Uusi‐Heikkilä et al., 2011). Consequently, shifting PMRNs in some cases could reflect contributions from phenotypic plasticity in response to unmeasured factors as opposed to being derived from underlying genetic change in response to harvest (Dieckmann & Heino, 2007; Kraak, 2007; Uusi‐Heikkilä et al., 2011). In theory, it may be possible to statistically account for variation in maturation attributable to additional sources of variation (Heino & Dieckmann, 2008; Heino, Dieckmann, & Godø, 2002), but this could be challenging because environmental conditions in Lake Erie are changing with respect to nutrient inputs, invasive species, and warming climate (Allinger & Reavie, 2013). Consistent with this line of reasoning, the highest standardized rates of phenotypic change in PMRN midpoint values calculated here (−0.9 to 28.2 kilodarwins; −0.1 to 2.4 haldanes) were higher than those reported in meta‐analyses of harvested fish stocks elsewhere (−57.6 to 26.5 kilodarwins; −1.9 to 1.2 haldanes; Darimont et al., 2009; Devine et al., 2012; Sharpe & Hendry, 2009). The rates we calculated for Lake Erie yellow perch are also higher than standardized rates reported for yellow perch in other Great Lakes (−1.18 to 1.78 haldanes; Feiner et al., 2015). We recommend that rapid changes in PMRN midpoint values or envelop widths over short time intervals be interpreted cautiously and that further exploration of nonevolutionary explanations for changes in the maturation of Lake Erie yellow perch is warranted.

This is one of few studies to have inferred the potential for evolved maturation responses for a commercially harvested fish in freshwaters. The size threshold for maturation assessed here as PMRN midpoints increased over a time period of 18–20 years, equivalent to five generations in yellow perch, at a time when fishing pressure was near to its lowest historic level. This suggests that reducing harvest to lower levels can select for delayed maturation in yellow perch, thus allowing for an evolutionary recovery of the population. Our study highlights the value that studies of commercially harvested freshwater fishes can have for understanding the generality of predictions about the direction and rate of fisheries‐induced evolution, the relative roles played by plastic and evolutionary mechanisms in shaping maturation in fishes, and how these mechanisms are likely influenced by the intensity and nature of harvesting.

## CONFLICT OF INTEREST

None declared.

## 5 DATA AVAILABILITY

Data for this study are available at: Data on exploitation rate are from Lake Erie Committee, Yellow Perch Task Group, http://glfc.org/annual-reports.php. Data for estimating PMRNs are from the Lake Erie partnership index fisheries survey database maintained by the Lake Erie Management Unit of the Ontario Ministry of Natural Resources and Forestry (OMNRF).

## References

[eva12764-bib-0001] Allendorf, F. W. , & Hard, J. J. (2009). Human‐induced evolution caused by unnatural selection through harvest of wild animals. Proceedings of the National Academy of Sciences of the United States of America, 106, 9987–9994. 10.1073/pnas.0901069106 19528656 PMC2702803

[eva12764-bib-0002] Allinger, L. E. , & Reavie, E. D. (2013). The ecological history of Lake Erie as recorded by the phytoplankton community. Journal of Great Lakes Research, 39, 365–382. 10.1016/j.jglr.2013.06.014

[eva12764-bib-0003] Baldwin, N. A. , Saalfeld, R. W. , Dochoda, M. R. , Buettner, H. J. , & Eshenroder, R. L. (2009). Commercial fish production in the Great Lakes 1867–2006. Retrieved from http://www.glfc.org/databases/commercial/commerc.php

[eva12764-bib-0004] Barot, S. , Heino, M. , O’Brien, L. , & Dieckmann, U. (2004). Estimating reaction norms for age and size at maturation when age at first reproduction is unknown. Evolutionary Ecology Research, 6, 659–678.

[eva12764-bib-0005] Belore, M. , Cook, A. , Hartman, T. , Hosack, M. , Kayle, K. , Knihht, C. , … Witzel, L. (2014). Report of the Lake Erie yellow perch task group. Ann Arbor, MI, USA.

[eva12764-bib-0006] Belore, M. , Cook, H. A. , Einhouse, D. , Hartman, T. , Kayle, K. A. , Macdougall, T. , … Witzel, L. (2016). Report of the Lake Erie yellow perch task group. Ann Arbor, MI, USA.

[eva12764-bib-0007] Brenden, T. O. , Brown, R. W. , Ebener, M. P. , Reid, K. , & Newcomb, T. J. (2013). Great Lakes commercial fisheries: Historical overview and prognosis for the future In Taylor W. W., Lynch A. J., & Leonard N. J. (Eds.), Great Lakes fisheries policy and management (pp. 339–397). East Lansing, MI: Michigan State University Press.

[eva12764-bib-0008] Darimont, C. T. , Carlson, S. M. , Kinnison, M. T. , Paquet, P. C. , Reimchen, T. E. , & Wilmers, C. C. (2009). Human predators outpace other agents of trait change in the wild. Proceedings of the National Academy of Sciences of the United States of America, 106, 952–954. 19139415 10.1073/pnas.0809235106 PMC2630061

[eva12764-bib-0009] Devine, J. A. , & Heino, M. (2011). Investigating the drivers of maturation dynamics in Barents Sea haddock (*Melanogrammus aeglefinus*). Fisheries Research, 110, 441–449. 10.1016/j.fishres.2011.05.016

[eva12764-bib-0010] Devine, J. A. , Wright, P. J. , Pardoe, H. E. , & Heino, M. (2012). Comparing rates of contemporary evolution in life‐history traits for exploited fish stocks. Canadian Journal of Fisheries and Aquatic Sciences, 69, 1105–1120. 10.1139/f2012-047

[eva12764-bib-0011] Diaz Pauli, B. , & Heino, M. (2013). The importance of social dimension and maturation stage for the probabilistic maturation reaction norm in *Poecilia reticulata* . Journal of Evolutionary Biology, 26, 2184–2196. 23937558 10.1111/jeb.12215

[eva12764-bib-0012] Dieckmann, U. , & Heino, M. (2007). Probabilistic maturation reaction norms: Their history, strengths, and limitations. Marine Ecology Progress Series, 335, 253–269. 10.3354/meps335253

[eva12764-bib-0013] Dieckmann, U. , Heino, M. , & Rijnsdorp, A. D. (2009). The dawn of Darwinian fishery management. ICES Insight, 46, 34–43.

[eva12764-bib-0014] Dunlop, E. S. , Eikeset, A. M. , & Stenseth, N. C. (2015). From genes to populations: How fisheries‐induced evolution alters stock productivity. Ecological Applications, 25, 1860–1868. 10.1890/14-1862.1 26591452

[eva12764-bib-0015] Dunlop, E. S. , Enberg, K. , Jørgensen, C. , & Heino, M. (2009). Toward Darwinian fisheries management. Evolutionary Applications, 2, 245–259. 25567878 10.1111/j.1752-4571.2009.00087.x PMC3352496

[eva12764-bib-0016] Dunlop, E. S. , Feiner, Z. S. , & Höök, T. O. (2018). Potential for fisheries‐induced evolution in the Laurentian Great Lakes. Journal of Great Lakes Research, 44, 735–747. 10.1016/j.jglr.2018.05.009

[eva12764-bib-0017] Dunlop, E. S. , Heino, M. , & Dieckmann, U. (2009). Eco‐genetic modeling of contemporary life‐history evolution. Ecological Applications, 19, 1815–1834. 10.1890/08-1404.1 19831072

[eva12764-bib-0018] Dunlop, E. S. , Shuter, B. J. , & Dieckmann, U. (2007). Demographic and evolutionary consequences of selective mortality: Predictions from an eco‐genetic model for smallmouth bass. Transactions of the American Fisheries Society, 136, 749–765. 10.1577/T06-126.1

[eva12764-bib-0019] Dunlop, E. S. , Shuter, B. J. , & Ridgway, M. S. (2005). Isolating the influence of growth rate on maturation patterns in the smallmouth bass (*Micropterus dolomieu*). Canadian Journal of Fisheries and Aquatic Sciences, 62, 844–853.

[eva12764-bib-0020] Eikeset, A. M. , Dunlop, E. S. , Heino, M. , Storvik, G. , Stenseth, N. C. , & Dieckmann, U. (2016). Roles of density‐dependent growth and life history evolution in accounting for fisheries‐induced trait changes. Proceedings of the National Academy of Sciences of the United States of America, 113(52), 15030–15035. 10.1073/pnas.1525749113 27940913 PMC5206539

[eva12764-bib-0021] Eikeset, A. M. , Richter, A. , Dunlop, E. S. , Dieckmann, U. , & Stenseth, N. C. (2013). Economic repercussions of fisheries‐induced evolution. Proceedings of the National Academy of Sciences of the United States of America, 110, 12259–12264. 10.1073/pnas.1212593110 23836660 PMC3725113

[eva12764-bib-0022] Enberg, K. , Jørgensen, C. , Dunlop, E. S. , Heino, M. , & Dieckmann, U. (2009). Implications of fisheries‐induced evolution for stock rebuilding and recovery. Evolutionary Applications, 2, 394–414. 25567888 10.1111/j.1752-4571.2009.00077.x PMC3352485

[eva12764-bib-0023] Enberg, K. , Jørgensen, C. , Dunlop, E. S. , Varpe, Ø. , Boukal, D. S. , Baulier, L. , … Heino, M. (2012). Fishing‐induced evolution of growth: Concepts, mechanisms and the empirical evidence. Marine Ecology, 33, 1–25. 10.1111/j.1439-0485.2011.00460.x

[eva12764-bib-0024] Farmer, T. M. , Marschall, E. A. , Dabrowski, K. , & Ludsin, S. A. (2015). Short winters threaten temperate fish populations. Nature Communications, 6, 1–10. 10.1038/ncomms8724 PMC4518244 26173734

[eva12764-bib-0025] Feiner, Z. S. , Chong, S. C. , Knight, C. T. , Lauer, T. E. , Thomas, M. V. , Tyson, J. T. , & Höök, T. O. (2015). Rapidly shifting maturation schedules following reduced commercial harvest in a freshwater fish. Evolutionary Applications, 8, 724–737. 10.1111/eva.12285 26240608 PMC4516423

[eva12764-bib-0026] Gingerich, P. (2001). Rates of evolution on the time scale of the evolutionary process. Genetica, 112–113, 127–144. 11838762

[eva12764-bib-0027] Gíslason, D. , McLaughlin, R. L. , Robinson, B. W. , Cook, A. , & Dunlop, E. S. (2017). Rapid changes in age and size at maturity in Lake Erie yellow perch (*Perca flavescens*) are not explained by harvest. Canadian Journal of Fish and Aquatic Sciences, 223, 211–223.

[eva12764-bib-0028] Grift, R. E. , Heino, M. , Rijnsdorp, A. D. , Kraak, S. B. M. , & Dieckmann, U. (2007). Three‐dimensional maturation reaction norms for North Sea plaice. Marine Ecology Progress Series, 334, 213–224. 10.3354/meps334213

[eva12764-bib-0029] Grift, R. E. , Rijnsdorp, A. D. , Barot, S. , Heino, M. , & Dieckmann, U. (2003). Fisheries‐induced trends in reaction norms for maturation in North Sea plaice. Marine Ecology Progress Series, 257, 247–257. 10.3354/meps257247

[eva12764-bib-0030] Haugen, T. , & Vøllestad, L. A. (2001). A century of life‐history evolution in grayling. Genetica, 112–113, 475–491. 11838784

[eva12764-bib-0031] Hauser, L. , Adcock, G. J. , Smith, P. J. , Bernal Ramirez, J. H. , & Carvalho, G. R. (2002). Loss of microsatellite diversity and low effective population size in an overexploited population of New Zealand snapper (*Pagrus auratus*). Proceedings of the National Academy of Sciences of the United States of America, 99, 11742–11747. 10.1073/pnas.172242899 12185245 PMC129339

[eva12764-bib-0032] Heino, M. , Díaz Pauli, B. , & Dieckmann, U. (2015). Fisheries‐induced evolution. Annual Review of Ecology, Evolution, and Systematics, 46, 461–480. 10.1146/annurev-ecolsys-112414-054339

[eva12764-bib-0033] Heino, M. , & Dieckmann, U. (2008). Detecting fisheries‐induced life‐history evolution: An overview of the reaction‐norm approach. Bulletin of Marine Science, 83, 69–93.

[eva12764-bib-0034] Heino, M. , Dieckmann, U. , & Godo, O. R. (2002). Estimating reaction norms for age and size at maturation with reconstructed immature size distributions: A new technique illustrated by application to Northeast Arctic cod. ICES Journal of Marine Science, 59, 562–575. 10.1006/jmsc.2002.1192

[eva12764-bib-0035] Heino, M. , Dieckmann, U. , & Godø, O. R. (2002). Measuring probabilistic reaction norms for age and size at maturation. Evolution, 56, 669–678. 10.1111/j.0014-3820.2002.tb01378.x 12038525

[eva12764-bib-0036] Heino, M. , & Godø, O. R. (2002). Fisheries‐induced selection pressures in the context of sustainable fisheries. Bulletin of Marine Science, 70, 639–656.

[eva12764-bib-0037] Hoarau, G. , Boon, E. , Jongma, D. N. , Ferber, S. , Palsson, J. , Van der Veer, H. W. , … Olsen, J. L. (2005). Low effective population size and evidence for inbreeding in an overexploited flatfish, plaice (*Pleuronectes platessa* L.). Proceedings of the Royal Society of London, Series B: Biological Sciences, 272, 497–503. 15799945 10.1098/rspb.2004.2963 PMC1578705

[eva12764-bib-0038] Hutchings, J. A. (2009). Avoidance of fisheries‐induced evolution: Management implications for catch selectivity and limit reference points. Evolutionary Applications, 2, 324–334. 25567884 10.1111/j.1752-4571.2009.00085.x PMC3352487

[eva12764-bib-0039] Hutchinson, W. F. , van Oosterhout, C. , Rogers, S. I. , & Carvalho, G. R. (2003). Temporal analysis of archived samples indicates marked genetic changes in declining North Sea cod (*Gadus morhua*). Proceedings of the Royal Society of London, Series B: Biological Sciences, 270, 2125–2132. 14561275 10.1098/rspb.2003.2493 PMC1691486

[eva12764-bib-0040] Jørgensen, C. , Ernande, B. , & Fiksen, Ø. (2009). Size‐selective fishing gear and life history evolution in the Northeast Arctic cod. Evolutionary Applications, 2, 356–370. 25567886 10.1111/j.1752-4571.2009.00075.x PMC3352490

[eva12764-bib-0041] Jørgensen, C. , Enberg, K. , Dunlop, E. S. , Arlinghaus, R. , Boukal, D. S. , Brander, K. , … Rijnsdorp, A. D. (2007). Managing evolving fish stocks. Science, 318, 1247–1248. 18033868 10.1126/science.1148089

[eva12764-bib-0042] Jørgensen, T. (1990). Long‐term changes in age at sexual maturity of Northeast Arctic cod (*Gadus morhua* L.). ICES Journal of Marine Science, 46, 235–248. 10.1093/icesjms/46.3.235

[eva12764-bib-0043] Kokkonen, E. , Vainikka, A. , & Heikinheimo, O. (2015). Probabilistic maturation reaction norm trends reveal decreased size and age at maturation in an intensively harvested stock of pikeperch *Sander lucioperca* . Fisheries Research, 167, 1–12. 10.1016/j.fishres.2015.01.009

[eva12764-bib-0044] Kraak, S. B. M. (2007). Does the probabilistic maturation reaction norm approach disentangle phenotypic plasticity from genetic change? Marine Ecology Progress Series, 335, 295–300.

[eva12764-bib-0045] Kuparinen, A. , & Hutchings, J. A. (2012). Consequences of fisheries‐induced evolution for population productivity and recovery potential. Proceedings of the Royal Society of London, Series B: Biological Science, 279, 2571–2579. 10.1098/rspb.2012.0120 PMC3350703 22398166

[eva12764-bib-0046] Kuparinen, A. , Kuikka, S. , & Merilä, J. (2009). Estimating fisheries‐induced selection: Traditional gear selectivity research meets fisheries‐induced evolution. Evolutionary Applications, 2, 234–243. 10.1111/j.1752-4571.2009.00070.x 25567864 PMC3352371

[eva12764-bib-0047] Kuparinen, A. , & Merilä, J. (2007). Detecting and managing fisheries‐induced evolution. Trends in Ecology and Evolution, 22, 652–659. 10.1016/j.tree.2007.08.011 17981361

[eva12764-bib-0048] Kuparinen, A. , Stenseth, N. C. , & Hutchings, J. A. (2014). Fundamental population‐productivity relationships can be modified through density‐dependent feedbacks of life‐history evolution. Evolutionary Applications, 7, 1218–1225. 10.1111/eva.12217 25558282 PMC4275093

[eva12764-bib-0049] Law, R. , & Grey, D. R. (1989). Evolution of yields from populations with age‐specific cropping. Evolutionary Ecology, 3, 343–359. 10.1007/BF02285264

[eva12764-bib-0050] Marty, L. , Dieckmann, U. , & Ernande, B. (2015). Fisheries‐induced neutral and adaptive evolution in exploited fish populations and consequences for their adaptive potential. Evolutionary Applications, 8, 47–63. 10.1111/eva.12220 25667602 PMC4310581

[eva12764-bib-0051] Mollet, F. M. , Kraak, S. B. M. , & Rijnsdorp, A. D. (2007). Fisheries‐induced evolutionary changes in maturation reaction norms in North Sea sole *Solea solea* . Marine Ecology Progress Series, 351, 189–199. 10.3354/meps07138

[eva12764-bib-0052] Mollet, F. M. , Poos, J. J. , Dieckmann, U. , & Rijnsdorp, A. D. (2016). Evolutionary impact assessment of the North Sea plaice fishery. Canadian Journal of Fish and Aquatic Sciences, 73, 1126–1137. 10.1139/cjfas-2014-0568

[eva12764-bib-0053] Morita, K. , & Fukuwaka, M.‐A. (2006). Does size matter most? The effect of growth history on probabilistic reaction norm for salmon maturation. Evolution, 60, 1516–1521. 10.1111/j.0014-3820.2006.tb01230.x 16929668

[eva12764-bib-0054] Morita, K. , Tsuboi, J. , & Nagasawa, T. (2009). Plasticity in probabilistic reaction norms for maturation in a salmonid fish. Biology Letters, 5, 628–631. 10.1098/rsbl.2009.0290 19493875 PMC2781952

[eva12764-bib-0055] Nagelkerke, N. J. D. (1991). A note on a general definition of the coefficient of determination. Biometrica, 78, 691–692. 10.1093/biomet/78.3.691

[eva12764-bib-0056] Olsen, E. M. , Heino, M. , Lilly, G. R. , Morgan, M. J. , Brattey, J. , Ernande, B. , & Dieckmann, U. (2004). Maturation trends indicative of rapid evolution preceded the collapse of northern cod. Nature, 428, 932–935. 10.1038/nature02430 15118724

[eva12764-bib-0057] Olsen, E. M. , Lilly, G. R. , Heino, M. , Morgan, M. J. , Brattey, J. , & Dieckmann, U. (2005). Assessing changes in age and size at maturation in collapsing populations of Atlantic cod (*Gadus morhua*). Canadian Journal of Fisheries and Aquatic Sciences, 62, 811–823.

[eva12764-bib-0058] OMNRF and OCFA (2016). 2016 Lake Erie partnership index fishing, project description and sampling protocol. Ontario Ministry of Natural Resources and Forestry.

[eva12764-bib-0059] Poste, A. E. , Hecky, R. E. , & Guildford, J. (2011). Evaluating microcystin exposure risk through fish consumption. Environmental Science and Technology, 45, 5806–5811. 10.1021/es200285c 21671629 PMC3148776

[eva12764-bib-0060] R Core Team (2014). R: A language and environment for statistical computing. Vienna, Austria: R Foundation for Statistical Computing Retrieved from http://www.r-project.org/

[eva12764-bib-0061] Rijnsdorp, A. D. (1993). Fisheries as a large‐scale experiment on life‐history evolution: Disentangling phenotypic and genetic effects in changes in maturation and reproduction of North Sea plaice, *Pleuronectes platessa*, L. Oecologia, 96, 391–401. 10.1007/BF00317510 28313655

[eva12764-bib-0062] Ruzzante, D. E. , Taggart, C. T. , Doyle, R. W. , & Cook, D. (2001). Stability in the historical pattern of genetic structure of Newfoundland cod (*Gadus morhua*) despite the catastrophic decline in population size from 1964 to 1994. Conservation Genetics, 2, 257–269.

[eva12764-bib-0063] Sepulveda‐Villet, O. J. , Stepien, C. A. , & Vinebrooke, R. (2011). Fine‐scale population genetic structure of the yellow perch *Perca flavescens* in Lake Erie. Canadian Journal of Fisheries and Aquatic Sciences, 68, 1435–1453.

[eva12764-bib-0064] Sharpe, D. M. T. , & Hendry, A. P. (2009). Life history change in commercially exploited fish stocks: An analysis of trends across studies. Evolutionary Applications, 2, 260–275. 25567879 10.1111/j.1752-4571.2009.00080.x PMC3352497

[eva12764-bib-0065] Sullivan, T. J. , & Stepien, C. A. (2015). Temporal population genetic structure of yellow perch spawning groups in the lower Great Lakes. Transactions of the American Fisheries Society, 144, 211–226. 10.1080/00028487.2014.982260

[eva12764-bib-0066] Swain, D. P. , Sinclair, A. F. , & Hanson, J. M. (2007). Evolutionary response to size‐selective mortality in an exploited fish population. Proceedings of the Royal Society of London, Series B: Biological Sciences, 274, 1015–1022. 17264058 10.1098/rspb.2006.0275 PMC2124474

[eva12764-bib-0067] Therkildsen, N. O. , Nielsen, E. E. , Swain, D. P. , & Pedersen, J. S. (2010). Large effective population size and temporal genetic stability in Atlantic cod (*Gadus morhua*) in the southern Gulf of St. Lawrence. Canadian Journal of Fisheries and Aquatic Sciences, 67, 1585–1595. 10.1139/F10-084

[eva12764-bib-0068] Trippel, E. A. (1995). Age at maturity as a stress indicator in Fisheries. BioScience, 45, 759–771. 10.2307/1312628

[eva12764-bib-0069] Uusi‐Heikkilä, S. , Kuparinen, A. , Wolter, C. , Meinelt, T. , O’Toole, A. C. , & Arlinghaus, R. (2011). Experimental assessment of the probabilistic maturation reaction norm: condition matters. Proceedings of the Royal Society of London, Series B: Biological Science, 278, 709–717. 10.1098/rspb.2010.1507 PMC3030844 20861054

[eva12764-bib-0070] van Walraven, L. , Mollet, F. M. , van Damme, C. J. G. , & Rijnsdorp, A. D. (2010). Fisheries‐induced evolution in growth, maturation and reproductive investment of the sexually dimorphic North Sea plaice (*Pleuronectes platessa* L.). Journal of Sea Research, 64, 85–93. 10.1016/j.seares.2009.07.003

[eva12764-bib-0071] Wang, H.‐Y. , Höök, T. O. , Ebener, M. P. , Mohr, L. C. , & Schneeberger, P. J. (2008). Spatial and temporal variation of maturation schedules of lake whitefish (*Coregonus clupeaformis*) in the Great Lakes. Canadian Journal of Fisheries and Aquatic Sciences, 65, 2157–2169. 10.1139/F08-124

[eva12764-bib-0072] Winemiller, K. O. , & Rose, K. A. (1992). Patterns of life‐history diversification in North American fishes: Implications for population regulation. Canadian Journal of Fisheries and Aquatic Sciences, 49, 2196–2218. 10.1139/f92-242

